# Household Conflicts with Snow Leopard Conservation and Impacts from Snow Leopards in the Everest and Annapurna Regions of Nepal

**DOI:** 10.1007/s00267-022-01653-4

**Published:** 2022-05-06

**Authors:** Jonathan H. Hanson

**Affiliations:** 1 Department of Geography, University of Cambridge, Cambridge, UK; 2 Jubilee Community Benefit Society, Larne, UK; 3 Snow Leopard Conservancy, Sonoma, CA USA

**Keywords:** Human-wildlife conflict, Annapurna conservation area, Sagarmatha national park, South asia, Human-wildlife coexistence, Carnivores

## Abstract

Impacts on households from large carnivores are frequently reported in the conservation literature, but conflicts between households and large carnivore conservation are not. Employing a human-wildlife coexistence framework that distinguishes between human-wildlife impacts on one hand, and human-conservation conflicts on the other, this paper presents data from Annapurna Conservation Area and Sagarmatha (Everest) National Park, Nepal, each with different models of conservation governance. Using systematic sampling, quantitative information from 705 households was collected via questionnaires, while 70 semi-structured interviews were conducted with key informants for cross-methods triangulation. 7.7% of households reported conflicts with snow leopard conservation in the previous 12 months, primarily due to damage to livelihoods; these were significantly higher in the Annapurna region. 373 livestock were reported lost by households to snow leopards in the previous 12 months, representing 3.4% of total livestock owned and US$ 132,450 in financial value. Livestock losses were significantly lower in the Everest area. In linear regression models, total household livestock losses to all sources best explained conflicts with snow leopard conservation and household livestock losses to snow leopards but the models for the former dependent variable had very low explanatory power. Conservation in general, and large carnivore conservation in particular, should distinguish carefully between impacts caused by coexistence with these species and conflicts with conservation actors and over the methods and interventions used to conserve carnivores, especially where these negatively impact local livelihoods. In addition, livestock husbandry standards are highlighted again as an important factor in the success of carnivore conservation programmes.

## Introduction

Negative physical interactions between wildlife and people have often been termed ‘human-wildlife conflict’ (Marchini 2014). However, the phrase may have less to do with the potential of mostly large wild mammals for causing damage and more to do with their power to evoke polarised opinions amongst different stakeholder groups (Linnell et al. 2005; Rastogi et al. 2012). Employing a human-wildlife coexistence (HWC) framework, conservationists should distinguish between human-wildlife impacts, on one hand, and human-conservation conflicts on the other (Redpath et al. 2015). It is also necessary to be more explicit about the different stakeholders with an interest in wildlife, of which conservation is but one, and the trade-offs between them often involved in finding a management compromise for HWC (McShane et al. 2011). This distinction between impacts and conflicts has also been made explicitly for snow leopards *Panthera uncia* (Mishra et al. 2016).

The environmental dimensions of human-wildlife impacts have generally received more attention (Inskip and Zimmerman 2009; Rastogi et al. 2012; Ripple et al. 2014). Social factors, however, have been subject to more research attention over the past decade. These include: crop and livestock losses, exacerbated by increasing human and animal populations (Mishra 1997; Namgail et al. 2007); poverty (Inskip et al. 2013); livelihood insecurity and uniformity (Ikeda 2004; Hemson et al. 2009; Dickman 2010); economic inequality (Dickman et al. 2011); gender inequality (Ogra and Badola 2008); a lack of appropriate fora for community management of human-wildlife impacts (Gurung et al. 2008); and husbandry practices (Jackson and Wangchuk 2001; Suryawanshi et al. 2013). Social spaces can also influence human-wildlife impacts. Impacts can occur in and around Protected Areas (PAs), especially where these act as sources for populations of large, and potentially destructive, mammal species (Karanth and Nepal 2012; Karanth et al. 2013). Impacts can also occur in social spaces outside of PAs, such as wildlife corridors (Nyhus and Tilson 2004; Nepal and Spiteri 2011).

Snow leopard predation on livestock is endemic across its range (Jackson et al. 2010). Reported rates of livestock predation by the species have included 11.1% in western Nepal (Devkota et al. 2013), 10.6% in central China (Li et al. 2013), 12.6% also in central China (Alexander et al. 2015), and 19.0% in Annapurna Conservation Area (ACA), Nepal (Ale et al. 2014). Consequently, human-snow leopard impacts are recognised as an important focal area for research, policy and practice, both rangewide and in Nepal (McCarthy and Chapron 2003; GSLEP 2013; Jackson et al. 2013; WWF 2015; DNPWC 2017). Yet, due in part to conservation’s ontological bias towards natural science, including in felid conservation (Rastogi et al. 2012; Ghosal et al. 2013), they have received limited scholarly attention. There is therefore an ongoing need for comprehensive social analysis of human-wildlife impacts in relation to snow leopards, as well as how it relates to knowledge, attitudes, access, influence and snow leopard conservation (Rashid et al. 2020).

Much ‘conflict’ between humans and wildlife is more precisely a form of conflict between humans and wildlife conservation (Linnell et al. 2005; Redpath et al. 2015). Increasingly, the importance of understanding the socio-economic and cultural context of such relationships, rather than just ascribing them to ecological influences or prescribing technical management solutions for them, has been recognised (Mehta and Heinen 2001; Olsson et al. 2004; Rust et al. 2016). A HWC perspective like this can present a clearer picture of the influences on human-conservation conflicts. Yet, as with attitudes to snow leopard conservation compared to attitudes to snow leopards (Hanson et al. 2019), there is a relative dearth of research on human-conservation conflicts compared to human-wildlife impacts.

As with human-wildlife impacts, livelihoods can be an important predictor of conflict between people and conservation (Adams and Hutton 2007) as can the presence of PAs (Khan and Bhagwat 2010). In fact, park-people relationships are one area of conservation conflict that have received more attention, especially in the Global South (Adams and Hutton 2007), including Nepal (Mehta and Heinen 2001; Nepal and Spiteri 2011; Karanth and Nepal 2012; Parker and Thapa 2012). Both problems and successes can exist with compensation, insurance and incentive schemes set up to mitigate both human-conservation conflicts and human-wildlife impacts in and around PAs (Dickman et al. 2011; Alexander et al. 2021). Conflicts can also occur within centralised and community-based conservation (CBC) governance settings (Bajracharya et al. 2006).

Although formal complaints to park authorities about livestock depredations were found to be minimal in the Annapurna and Everest regions of Nepal (Jackson et al. 1996; Ale et al. 2007), neighbouring regions provide additional details. Widespread dissatisfaction with the livestock compensation scheme in Qomolongma National Park, adjoining Sagarmatha National Park (SNP) on the Tibetan side, has been reported (Chen et al. 2016). In Nepal’s eastern Kanchenjunga region, livestock herders were found to be mostly negative towards snow leopard conservation policy (Ikeda 2004). Whether for conservation conflicts or for wildlife impacts, it is clear that a more nuanced approach that integrates data on livelihoods, governance, knowledge and attitudes is essential (Olsson et al. 2004), including for snow leopards (Rashid et al. 2020).

Employing a HWC framework that distinguishes between human-wildlife impacts on one hand, and human-conservation conflicts on the other, the study, therefore, asked the following research questions:

1. What are household conflicts with snow leopard conservation and what factors best explain these?
2. What are household impacts from snow leopards and what factors best explain these?

## Methods

### Ethical Approval

Ethical approval was provided by the Ethics Review Group at the University of Cambridge’s Department of Geography. Field research approval was provided by the National Trust for Nature Conservation, Nepal, and the Department of National Parks and Wildlife Conservation, Nepal.

### Study Areas

**Fig. 1:**
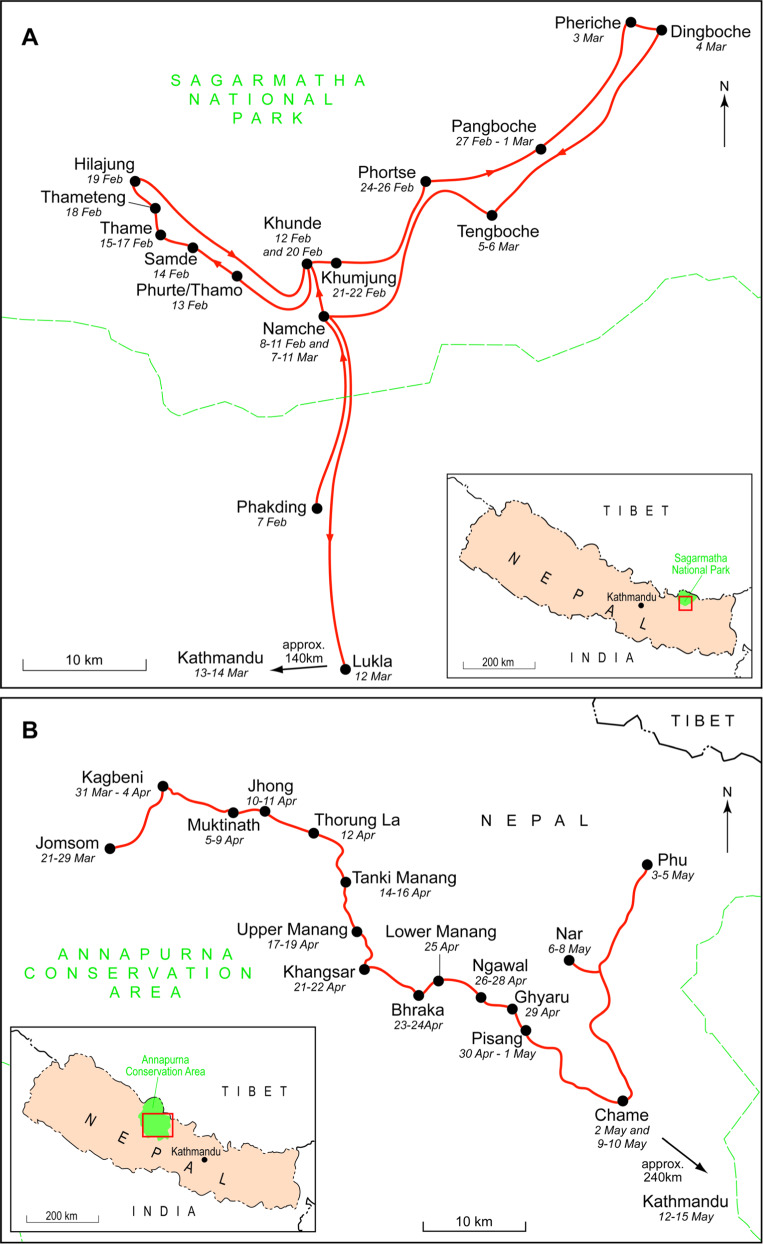
Study areas in Nepal showing areas and dates sampled. **A** Sagarmatha National Park. **B** Annapurna Conservation Area. Locations outside of study sites, and the dates visited, shown for illustrative purposes only

The ACA is a Protected Area (PA) in the mid-western region of Nepal, covering over 7629 km² of Himalayan landscape (Fig. 1). Elevations range from 1000 m to over 8000 m. Snow leopards are present at a range of densities and primarily prey on blue sheep *Pseudois nayaur* (Ale et al. 2014). A co-management governance approach is employed, shared between Conservation Area Management Committees, representing local communities, and the National Trust for Nature Conservation (NTNC), a Nepali NGO. More than 100,000 people reside in the ACA (Government of Nepal 2012), deriving their income primarily from agro-pastoralism and tourism (Bajracharya et al. 2006). ACA is one of the world’s most popular trekking destinations and received an all-time high of 181,000 international tourists in 2019 (ACAP 2021). Visitor numbers to ACA fell sharply from 181,000 in 2019 to 18,796 in 2020 due to the COVID-19 pandemic and recovery to previous levels is likely to take several years (ACAP 2021).

Sagarmatha National Park (Fig. 1) was gazetted in 1976, with a buffer zone added in 2002 (Baral and Heinen 2005). Totaling 1148 km^2^, habitats in SNP vary between permanent snow at 8848 m and temperate oak and pine forests at 2845 m (Bhuju et al. 2007). Snow leopard habitat is largely characterised by sub-alpine and alpine vegetation between 3500 and 5500 m. Approximately 3500 people live in 63 settlements within the park (Government of Nepal 2012), engaged in a combination of agro-pastoralism and tourism (Brower 1996; Padoa-Schioppa and Baietto 2008). SNP has a more centralised conservation governance regime, though with increasing local devolution and involvement since the introduction of a buffer zone in 2002 (Daconto and Sherpa 2010).

### Questionnaire Preparation and Administration

The primary data gathering mechanism was a household questionnaire administered by Nepali research assistants in Nepali or English. Although the majority of the questionnaire comprised closed questions, some open questions were also added to assess respondents’ reasons for their conflicts with snow leopard conservation, a practice recommended for the use of questionnaires in ecological studies (White et al. 2005). As livestock are often owned and tended by family groups, the household was the main unit of analysis, a common trend in conservation social science assessments (Schreckenberg et al. 2010). A range of potential socio-economic explanatory variables was included in the questionnaire based on a literature review (Table 1). The two dependent variables measured were: a) self-reported number of household livestock killed by snow leopards in the last 12 months (log^10^ scale); b) self-reported number of household conflicts with snow leopard conservation in the last 12 months. A household questionnaire draft was then piloted with 24 households outside SNP and two Nepali-speaking research assistants were trained in its delivery. A copy of the questionnaire is included in the Supplementary Information.

**Table 1:** Household data measured by questionnaire and used as variables in linear models

Variable type	Data type	Variable
Dependent	Continuous	Self-reported number of household livestock killed by snow leopards in the last 12 months (computed as log^10^ scale)
		Self-reported number of household conflicts with snow leopard conservation in the last 12 months, as a total of number of household conflicts with: Park management; Local committee; Ban on the killing of snow leopards; Livestock compensation scheme; Corral construction; Environmental education activities; Limits on the collection of Non-Timber Forest Products (NTFPs); Limits on the collection of wood; Other
Independent	Continuous	Self-reported total household livestock owned in the last 12 months (computed as log^10^ scale)
		Self-reported number of livestock lost by household to all mortality sources in the last 12 months (computed as log^10^ scale)
		Self-reported number of household livestock killed by snow leopards in the last 12 months (computed as log^10^ scale)
		Self-reported number of household conflicts with snow leopard conservation in the last 12 months
		Household adult literacy rate
		Total household members
		Household Sustainable Livelihoods Index score (see Supplementary Information for more detail on this index and its computation)
	Binary	Study site/Protected Area/Household location
		Livestock as primary source of household financial income
		Tourism as primary source of household financial income
		Other source as primary source of household financial income
		Livestock as primary source of household financial income

Questionnaire data were collected from 260 households in SNP between February and March 2014 (Fig. 1). In ACA between March and May 2014, 445 household questionnaires were completed. The questionnaires were administered to a male or female Nepali research assistant in Nepali with a male or female adult within the household. Systematic sampling was used for the questionnaire due to the absence of a sampling frame for the settlements. Census data provided the number of households in each Village Development Committee/Gaunpalika and a quarter of these were sampled (Paudel and Thapa 2001). Each settlement was divided into two, with each research assistant collecting data in one-half only. Additionally, back-checking of 10% of household questionnaires by the Principal Investigator, as recommended by White et al. (2005). The response rate to the questionnaire was 96.2%, based on 733 household members invited to participate. The combined sample was 52.1% male and 47.9% female, and the mean respondent age was 42.66 ± 15.29.

### Questionnaire Data Analysis

For numbers of self-reported household livestock owned and numbers of self-reported household livestock lost to snow leopards (Table 1), data were changed to a log^10^ scale before inferential analysis due to significant variation within each variable, following Zimmermann et al. (2005). To test for inter-observer consistency between the two research assistants, independent *t*-tests were used (Field 2013). Descriptive and inferential statistics were used to analyse the quantitative data, with the multiple regression models being used for explanation rather than prediction (Mac Nally 2000). Prior to this, data were checked to ensure they met the necessary assumptions for multiple regression models—linearity, reliability, homoscedasticity and normality—and multicollinearity between variables did not exceed the recommended limit of 0.7 for Pearson’s’ correlation coefficient (Osborne and Waters 2002).

One set of linear regression models used ‘Number of household conflicts with snow leopard conservation’ as the dependent variable, while the other set used ‘Number of household livestock killed by snow leopards (log^10^ scale)’ (Table 1). Multiple regression models were constructed for combined samples and for each PA, except for household conflicts with snow leopard conservation in SNP, as there were not enough conflicts to allow regression analysis. Hierarchical entry based on theoretical suitability from the literature reviewed was used, instead of entry based on statistical significance alone, while the adjusted r-squared change results from the regression models were used to determine goodness-of-fit were used for model selection (Mac Nally 2002). As P-P plots to test for normality in multiple regression models indicated some evidence of non-normality in the dependent variable, bootstrapping was therefore used for the final models selected based on the adjusted r-squared change results (Field 2013).

### Key Informant Interviews

A semi-structured interview sheet, with a general structure mirroring that of the questionnaire, was also developed for the purposes of concurrent, cross-methods triangulation (Valentine 1997; Mikkelsen 2005). A copy of this is included in Supplementary Information. It comprised mostly of open questions, but also gathered quantitative data on current market valuations of livestock. Convenience and snowball sampling were used to identify key local informants, and the target sample set at 10% of the questionnaire sample. This resulted in 26 interviews in SNP and 44 in ACA.

The majority of interviews were administered in Nepali by two research assistants, with the PI always present. After transcription, the interview data were analysed both qualitatively and quantitatively to complement and triangulate the questionnaire analysis (Mikkelsen 2005). This follows Nepal and Spiteri’s (2011) similar approach with their analysis of livelihoods and conservation in the Makalu-Barun Conservation Area, neighbouring SNP. For instance, median valuations of livestock gathered in the key informant interviews were used to calculate the financial impact of snow leopard predation on households, while nuanced information on the causes of conflicts between snow leopard conservation and households was obtained.

## Results and Discussion

### Household Conflicts with Snow Leopard Conservation

Few studies have empirically assessed conflict between people and snow leopard conservation, despite it being recognised as an information gap for snow leopards (Rosen et al. 2012) and other large carnivores (Linnell et al. 2005; Rastogi et al. 2012). In this study, instances of conflict assessed included a range of local conservation actors and interventions, including: park management; local committees; a ban on the killing of snow leopards; livestock compensation schemes; corral construction; environmental education activities; limits on the collection of Non-Timber Forest Products; limits on the collection of wood; other interventions. Only 7.7% of households surveyed overall recorded conflicts in the previous 12 months. This confirms anecdotal evidence from ACA (Jackson et al. 1996) and SNP (Ale et al. 2007) that conflict with snow leopard conservation is relatively infrequent. Triangulation interviews, however, found considerably higher rates of conflict with 28.6%% of interviewees reporting conflicts with conservation actors and 37.1% with conservation interventions occurring in their locality (*N* = 70; Table 2). This suggests that actual or perceived cases of conflict may be under-reported by households.

**Table 2:** Community conflict with snow leopard conservation and compensation for livestock losses to snow leopards based on key informant interviews

Question	Response	Combined *N* = 70		SNP *N* = 26		ACA *N* = 44	
		*N*	%	*N*	%	*N*	%
Conflict with actors		20	28.6	3	11.5	17	38.6
Reason(s) for conflict with actors	[Sample size]	19		3		16	
	Lack of local benefits	1	5.3	1	33.3	0	0.00
	Livelihood damage	9	47.4	2	66.7	7	43.8
	Other	1	5.3	0	0.0	1	6.3
	Bureaucracy and livelihood damage	8	42.1	0	0.0	8	50.0
Conflict with interventions		26	37.1	11	42.3	15	34.1
Reason(s) for conflict with interventions	[Sample size]	26		11		15	
	Lack of local benefits	2	7.7	2	18.2	0	0.0
	Livelihood damage	18	69.2	7	63.6	11	73.3
	Other	1	3.8	3.8	9.1	0	0.0
	Bureaucracy and livelihood damage	5	19.2	3.8	9.1	4	26.7
Received compensation	No	33	68.8	19	100.0	14	48.3
	Yes	10	20.8	0	0.0	10	34.5
	Sometimes	5	10.4	0	0.0	5	17.2
	Total	48	100.0	19	100.0	29	100.0
Reason(s) for not receiving compensation	Bureaucracy	16	41.0	5	29.4	11	50.0
	Limited amount	3	7.7	0	0.0	3	13.6
	Not insured	2	5.1	0	0.00	2	9.1
	Scheme collapsed/irrelevant	3	7.7	1	5.9	2	9.1
	Haven’t reported/not aware of scheme	7	17.9	6	31.3	1	4.5
	>1 negative reason	8	20.5	5	29.4	3	13.6
	Total	39	100.0	17	100.0	22	100.0

Instances of household conflict with snow leopard conservation can also be compared across study sites. In SNP, 3.8% (*N* = 260) of households reported conflict with snow leopard conservation in the previous 12 months, while in ACA, 9.9% (*N* = 445) did so. The mean figure in the latter was significantly higher than in the former (*t*(643) = −2.44; *p* = ≤0.05). In part, this is due to the significantly higher rates of livestock losses to snow leopards in ACA (see section 3.3), which itself occurs due to differing socio-economic and ecological conditions in the two PAs (Ale et al. 2007; Bhuju et al. 2007; Ale et al. 2014). However, as conflict with conservation is also a product of conservation governance (Marchini 2014; Redpath et al. 2015), the higher rate in ACA may be due to perceived mismanagement of conservation in the PA’s decentralised co-management model (Teacher, ACA; Youth leader × 2, ACA; Community leader, ACA).

Based on the key informant interviews, household conflicts with snow leopard conservation were also broken down into conflicts with particular snow leopard conservation actors and specific snow leopard conservation interventions (*N* = 56), including park management (7.1%); local conservation committee (1.8%); a ban on killing snow leopards or their prey (3.6%); the livestock compensation scheme (64.3%); wood and Non-Timber Forest Product collection (7.1%); and more than one conflict (16.1%). With actors, there were more conflicts with PA authorities—the DNPWC in SNP and the NTNC in ACA—than there were with local conservation committees, a trend consistent with the literature on CBC (Bajracharya et al. 2006). However, the most frequent conflict types were related to various interventions, suggesting that household altercations with snow leopard conservation can be complex and multi-faceted, as found with other carnivore species in Namibia (Rust et al. 2016). Of these, the livestock compensation scheme was the most frequently cited element of snow leopard conservation that was cited as problematic.

The reasons for these household conflicts also varied, as additional data from the key informant interviews illustrates. They include (*N* = 56): a lack of local benefits (14.3%); damage to livelihoods (64.3%); bureaucratic complexity and delay (5.4%); other reasons (1.8%); and more than one reason (14.2%). As with types of conflicts, the relative frequency of >1 reason suggests that negative household interactions with snow leopard conservation can have more than one cause (Rust et al. 2016). However, damage to livelihoods is clearly the most common reason for these altercations, with almost two-thirds of respondents citing this in each PA. Triangulation interviews corroborated these findings (Table 2), with 87.5% of interviewees suggesting ‘livelihood damage’ and ‘bureaucracy and livelihood damage’ as the main reasons for conflicts with actors (*N* = 20) and 88.4% over interventions (*N* = 26). These observations are also consistent with the literature, particularly on the potential constraints of PAs on livelihoods (Adams and Hutton 2007; Khan and Bhagwat 2010; Karanth and Nepal 2012).

### Household Conflicts Over Livestock Compensation

Compensation for livestock losses to snow leopards was also analysed separately. Of the 111 households eligible for compensation in questionnaire responses, 93% had not, or not yet, received it. This is similar to findings in India (Karanth et al. 2013) and China (Alexander et al. 2015), with payment made in only 31% of cases in the Indian study. There was, however, no significant difference in the mean likelihood of compensation for livestock losses to snow leopards between SNP and ACA (*t* (117) = −1.09). This is despite the scheme being more comprehensive and better established in ACA, as compensation likelihood in triangulation interviews suggests (Supplementary Information 2). This may explain why ACA has significantly higher levels of household conflict with snow leopard conservation. For example, CBC may have effectively over-promised and under-delivered in ACA, resulting in heightened expectations of effective conservation solutions, such as compensation schemes, and greater disappointment when these fail, are perceived to have failed or suffered from any number of challenges. This may also explain the significantly less positive attitudes toward park management and to local conservation committees in ACA than in SNP (Hanson et al. 2019).

The most common reason for households not receiving compensation that triangulation interviews suggested was ‘bureaucracy’, cited by 41.0% of key informant interviewees (*N* = 39; Table 2). This has been a frequent critique of compensation schemes (Rosen et al. 2012; Chen et al. 2016). Yet the need for prompt payment has to be balanced with appropriate audits, checks and balances (Hemson et al. 2009; Alexander et al. 2021), a time-consuming process in itself, as several interviewees pointed out (Women’s leader, SNP; Microcredit cooperative officer, SNP; Teacher, ACA). The next most frequent reason was a multiple one, suggesting that the reasons for compensation schemes malfunctioning can be numerous and complex (Dickman et al. 2011).

### Explaining Household Conflicts with Snow Leopard Conservation

The social factors that explain human conflicts with snow leopard conservation, or with the conservation of other large carnivores, have received limited quantitative analysis to date. Here, 11 potential explanatory variables were tested for relationships with self-reported household conflicts with snow leopard conservation in a multivariate context (Supplementary Information 1). A linear model was not computed for the SNP sample as the number of households reporting conflicts with snow leopard conservation was too small (*n* = 10). The order of inclusion in the models was hierarchical and theoretical, and based on similar analyses in other published studies of human-wildlife impacts (Karanth et al. 2013; Suryawanshi et al. 2013), due to the relative absence of empirical analyses of predictors of human-conservation conflicts. Additional and diagnostic information for each model is contained in Supplementary Information 2 and 3.

In both models, total number of livestock lost to all source of mortality, whether snow leopards, other predators, disease, accidents, bad weather and various other causes, was the only explanatory variable that was significant. It explained 4.5% of the variation in ACA (*R*² = 0.045; *b* = 0.21 [0.039, 0.37]; *p* = 0.019) and only 4% overall (*R*² = 0.040; *b* = 0.17 [0.028, 0.071]; *p* = 0.019), meaning caution should be exercised in extrapolating from these models. Nevertheless, the total numbers of livestock lost to all source of mortality was the only significant variable, and using this as a proxy for husbandry standards, this relationship underscores the significance of livestock management approaches for carnivore, and snow leopard, conservation noted elsewhere (Kolowski and Holekamp 2006; Wang and Macdonald 2006; Namgail et al. 2007; Jackson et al. 2010). There is, however, a lack of quantitative empirical studies of human-conservation conflicts with which to compare, though a qualitative analysis of Namibian livestock and game farms did find a link between husbandry standards and human-conservation conflicts at the intra-farm level (Rust et al. 2016).

It also adds to the body of knowledge on the impact of livelihood factors in contributing to human-conservation conflicts (Adams and Hutton 2007; Rust et al. 2016). However, the relative explanatory weakness of these models in explaining household conflicts with snow leopard conservation suggests that environmental factors, such as snow leopard and snow leopard prey densities, rather than social factors may be the main drivers here. Additional research is required.

### Household Livestock Losses

Of the studies that have analysed livestock depredation rates by snow leopards, only some have also reported total herd losses per annum. In this study, the total of self-reported losses to all sources of mortality across all livestock classes gave an annual herd loss of 9.3% (Table 3). These levels are similar to the level reported in the literature (Devkota et al. 2013; Li et al. 2013; Alexander et al. 2015), with the exception of Ale et al. (2014).

**Table 3:** Self-reported household livestock losses in total and to snow leopards in the previous 12 months

	Livestock class	Combined *N* = 705				SNP *N* = 260				ACA *N* = 445				Difference *p = ≤ 0.05
		Median	Max.	Sum	Mean ± SD	Median	Max.	Sum	Mean ± SD	Median	Max.	Sum	Mean ± SD	
Total	Cattle	0	7	152	0.28 ± 0.79	0	5	47	0.28 ± 0.077	0	7	105	0.28 ± 0.80	*t* (703) = 0.093
	Sheep/goats	0	18	435	0.80 ± 2.31	0	0	0	0.00 ± 0.00	0	18	435	1.14 ± 2.70	*t* (579) = −8.27*
	Equines	0	3	38	0.07 ± 0.31	0	1	3	0.02 ± 0.13	0	3	35	0.09 ± 0.36	*t* (633) = −3.48*
	Yaks/yak hybrids	0	23	328	0.60 ± 2.27	0	20	103	0.61 ± 1.82	0	23	225	0.59 ± 2.44	*t* (703) = 0.10
	Other	0	10	64	0.12 ± 0.82	0	0	0	0.00 ± 0.00	0	10	64	0.17 ± 0.98	*t* (579) = −3.36*
	Total	0	40	1017	1.38 ± 3.20	0	6	153	0.49 ± 1.00	0	40	864	1.90 ± 3.86	*t* (540) = −7.31*
	% of total herd	–	–	9.3	–	–	–	10.8	–	–	–	9.0	–	–
Snow leopards	Cattle	0	5	44	0.28 ± 0.76	0	3	19	0.61 ± 0.80	0	5	25	0.20 ± 0.73	*t* (443) = −2.61*
	Sheep/goats	0	12	107	0.69 ± 1.92	0	0	0	0.00 ± 0.00	0	12	107	0.86 ± 2.11	*t* (523) = 4.56*
	Equines	0	2	22	0.14 ± 0.42	0	1	3	0.09 ± 0.30	0	2	19	0.15 ± 0.44	*t* (703) = 0.72
	Yaks/yak hybrids	0	21	200	1.27 ± 2.97	0	9	30	0.97 ± 1.70	0	21	170	1.35 ± 3.20	*t* (489) = 0.91
	Other	0	0	0	0.00 ± 0.00	0	0	0	0.00 ± 0.00	0	0	0	0.00 ± 0.00	n/a
	Total	0	21	373	0.65 ± 2.10	0	9	52	0.26 ± 0.60	0	21	321	0.82 ± 2.46	*t* (475) = 4.16*
	% of total herd	–	–	3.4	–	–	–	3.7	–	–	–	3.36	–	–

Mean losses of livestock by households differed significantly between study sites for some livestock categories (Table 3). ACA suffered higher rates of loss of sheep/goats, equines and other livestock species. Meanwhile, rates of loss of cattle and yaks/yak hybrids were similar across the two sites. However, ACA experienced significantly higher levels of mean household livestock losses overall, probably because sheep and goats were phased out from SNP due to meet prevailing conservation policy (Bhuju et al. 2007), and also because snow leopard densities were higher in ACA (Ale et al. 2014; DNPWC 2017).

Only one of the snow leopard predation studies listed above examined the financial impact of livestock losses overall. The economic value of total livestock losses to households in their sample from Central China was US$ 6193 each over the previous 12 months (Li et al. 2013). This is considerably higher than the US$ 492 per herding household noted for SNP and ACA combined in this study (Table 4), with the median value for each livestock class based on quantitative data from triangulation interviews (*N* = 70). This difference may be due to higher average holdings of livestock in the study by Li et al. (2013), particularly of more valuable large-bodied stock, such as yaks.

**Table 4:** Household livestock losses in financial terms in total and to snow leopards in the previous 12 months

	Livestock class with median value (in US$) per animal in brackets	Combined *N* = 705		SNP *N* = 260		ACA *N* = 445	
		Lost	Total value (US$)	Lost	Total value (US$)	Lost	Total value (US$)
Total	Cattle (125)	152	19,000	47	5875	105	13,125
	Sheep/goats (150)	435	65,250	0	0	435	65,250
	Horses/mules/donkeys (950)	38	36,100	3	2850	35	33,250
	Yaks/yak hybrids (450)	328	147,600	103	46,350	225	101,250
	Total value lost	953	267,950	153	55,075	864	212,875
	Total value lost per household with livestock	–	492	–	338	–	557
Snow leopards	Cattle (125)	44	5500	19	2375	25	3125
	Sheep/ goats (150)	107	16,050	0	0	107	16,050
	Horses/mules/donkeys (950)	22	20,900	3	2850	19	18,050
	Yaks/yak hybrids (450)	200	90,000	30	13,500	170	76,500
	Total value lost	373	132,450	52	18,725	321	113,725
	Total value lost per household with livestock	–	243	–	115	–	298

Of the most important reasons for household livestock losses (*N* = 272), snow leopards were cited as the primary cause of livestock mortality in this study (33.1%), followed by disease (22.8%), other predators (17.6%), accidents (12.1%), weather (9.6%) and other (4.8%). While some other studies have confirmed this trend (Devkota et al. 2013), other studies have listed different factors, including predation by other carnivore species, as the main contributor to livestock losses (Li et al. 2013; Ale et al. 2014; Alexander et al. 2015). Of the other predatory species mentioned, common leopards in SNP and jackals in ACA were frequently reported in interviews (Teacher and microcredit cooperative officer, SNP; Conservation leader, SNP; Park officer, ACA; Buddhist lama, ACA).

### Household Impacts from Snow Leopards

The overall loss of livestock to snow leopards reported by households was 16.6%, comprising 11.5% in SNP and 18.0% in ACA. The annual overall loss to the species as a percentage of the total herd was 3.4% (Table 3), approximately a third of total losses, which is consistent with the proportion reporting the species as the primary cause of livestock loss. This figure is also the same as the mean of various studies in the literature, which reported annual livestock predation rates by snow leopards of between 0.3% and 12.0% (Mishra 1997; Jackson and Wangchuk 2001; Devkota et al. 2013; Li et al. 2013; Ale et al. 2014; Alexander et al. 2015; Chen et al. 2016). There appear to be no published estimates of livestock losses to snow leopards in SNP, apart from a figure of 1.9% estimated for the Phortse area (Ale et al. 2007).

When mean household livestock losses to snow leopards were compared and contrasted between SNP and ACA, the data showed two significant differences (Table 3): (i) killings of sheep/goats were higher in ACA, where significantly higher numbers occurred, (ii) while killings of cattle were significantly higher in SNP, even though there were significantly lower numbers of cattle in SNP than in ACA. The difference may be due to the absence of sheep/goats from SNP for conservation reasons (Bhuju et al. 2007) and the relatively low densities of Himalayan tahr (Ale et al. 2007), leading to increased snow leopard predation on cattle. Losses of yaks/yak hybrids and equines were not significantly different between the two study sites, even though ACA supports significantly higher numbers of equines.

The value of livestock losses to snow leopards was less evenly spread between the two sites than the value of livestock losses overall (Table 4), with a bias towards ACA. This was probably due to smaller populations of snow leopards and sheep/goats in SNP (Bhuju et al. 2007). The combined figure of US$ 243 worth of livestock losses per herding household in the previous 12 months is within the range of figures reported elsewhere. These included widely varying figures of US$ 33.80 in Upper Mustang, ACA (Aryal et al. 2014), US$ 128 in Ladakh, India (Mishra 1997) and US$ 646 in central China (Li et al. 2013).

Analysis of the spatial dimensions of livestock losses to snow leopards (*N* = 114) indicated a clear bias towards high pastures (71.1%), followed by low pastures (13.2%), cultivated land/settlements (13.2%), barren land (1.8%) and other (0.9%). The high pasture figure from questionnaires is also corroborated by triangulation interviews, which gave an estimate of 61.4%. The temporal dimensions of livestock losses to snow leopards (*N* = 110) showed that half of such killings took place during winter (50.0%), followed by Spring (14.5%), Summer (14.5%), Autumn (12.8%) and unsure (8.2%). This clear trend is reported elsewhere in the literature for Nepal (Devkota et al. 2013), including for ACA (Aryal et al. 2014). Triangulation interviews also confirmed winter as the key time for livestock kills by snow leopards in both ACA and SNP, with a reported figure of 50%.

### Explaining Household Impacts from Snow Leopards

The social factors which explain impacts on households from snow leopards, i.e. livestock losses, have been less considered than the ecological factors. In this study, 11 independent variables were therefore analysed for their potential role in explaining livestock losses to snow leopards, in a multivariate analyses (Supplementary Information 4). The order of inclusion in the multiple regression models was hierarchical and theoretical, based on similar modelling in other published studies (Hemson et al. 2009; Karanth et al. 2012; Suryawanshi et al. 2013). Additional and diagnostic information for each model is contained in Supplementary Information 5, 6 and 7.

Like with the multivariate analyses of factors best-explaining household conflicts with snow leopard conservation, total household livestock losses were also the only explanatory variable that was significant in each of the three multivariate models explaining livestock losses to snow leopards. It explained 43% of the variation in ACA (*R*² = 0.431; *b* = 0.49 [0.39, 0.59]; *p* = 0.001), 39% in SNP (*R*² = 0.388; *b* = 0.38 [0.26, 0.52]; *p* = 0.001) and 43% overall (*R*² = 0.430; *b* = 0.47 [0.37, 0.55]; *p* = 0.001). As discussed for human-conservation conflicts, this variable is used as a proxy for husbandry standards in this study. Various studies have also identified husbandry practices as a key concern for snow leopard conservation (Jackson et al. 2010; Ale et al. 2014; Chen et al. 2016; Mishra et al. 2016). The importance of this variable was approximately equal in both SNP and ACA, despite significantly lower livestock holdings in the former.

Yet where husbandry has been identified as a problem previously, herders were either unwilling to change their practices (Jackson and Wangchuk 2001) or perceived that predator population increases were to blame (Chen et al. 2016). In addition, the growth of tourism in snow leopard habitat may reduce the availability of labour for livestock guarding in both ACA (Ale et al. 2014) and SNP (Ale et al. 2007), as some interviewees also noted (Hotel owners, SNP; Buddhist monk, SNP; Teacher, SNP; Community leader, ACA).

## Conclusion

For both human-snow leopard impacts and human-conservation conflicts the strongest factor explaining incidents in both cases was the number of household livestock lost to all sources of mortality. Taken as a proxy for husbandry standards, this is consistent with numerous other studies (Jackson et al. 2010; Ale et al. 2014; Chen et al. 2016; Mishra et al. 2016). That ACA had significantly higher levels of impacts and conflicts than SNP is partly due to socio-economic and ecological differences, but also to the relationship between sectors of the community in ACA and park authorities, particularly regarding real or perceived conflicts with the actors and interventions involved in snow leopard conservation. The study therefore addresses one of the research gaps highlighted by Rashid et al. (2020) in their review of human-snow leopard coexistence research, namely exploring the socio-cultural context of such events. In addition, these results complement a growing body of knowledge that seeks to understand the delicate dynamics of human-conservation conflicts, as distinct from human-wildlife impacts (Redpath et al. 2015; Mishra et al. 2016), including with other large carnivores (Rastogi et al. 2012); with livestock compensation schemes for large carnivore conservation (Dickman et al. 2011; Alexander et al. 2021); and in and around PAs in Nepal (Mehta and Heinen 2001; Nepal and Spiteri 2011; Karanth and Nepal 2012; Parker and Thapa 2012)). Additional and nuanced research at these and other study sites is required to further explore these relationships with snow leopards and other large predators, with those who seek to conserve them and with the communities who live alongside them.

## Supplementary information

Supplementary Information
