# Supplementary material for: Household Conflicts with Snow Leopard Conservation and Impacts from Snow Leopards in the Everest and Annapurna Regions of Nepal
Source: Environ Manage. 2022 May 6;70(1):105–16. doi: 10.1007/s00267-022-01653-4 (PMC9075924; doi:10.1007/s00267-022-01653-4)
Supplement: Supplementary file 1 — Supplementary Information [file 267_2022_1653_MOESM1_ESM.docx]

**Supplementary Information 1.** Linear models explaining household conflicts with snow leopard conservation in previous 12 months for a combined sample and in ACA.

| **Model** | **Variable** | ***b*** | ***SE B*** | ***Standardised b*** | ***p*** |
| --- | --- | --- | --- | --- | --- |
| **Combined**  R² = .040  N = 705 | **Constant** | .050  (-.024, .14) | .042 | --- | p = .23 |
|  | **Study site**** | -.012  (-.063,.041) | .027 | -.016 | p = .69 |
|  | **Total household livestock (log¹º scale)** | -.004  (-.060, .051) | .028 | -.006 | p = .89 |
|  | **Household adult literacy rate** | -.040  (-.14, .046) | .048 | -.036 | p = .41 |
|  | **Number of livestock lost by household (log¹º scale)** | .17  (.028, .31) | .071 | .16 | p = .019 |
| **ACA**  R² = .045  N = 445 | **Constant** | -.010  (-.076, .071) | .036 | --- | p = .78 |
|  | **Total household livestock (log¹º scale)** | -.002  (-.078, .076) | .039 | -.003 | p = .95 |
|  | **Number of livestock lost by household (log¹º scale)** | .21  (.039, .37) | .083 | .20 | p = .019 |

Note. * 0 = no; 1 = yes. ** 0 = SNP; 1 = ACA.

**Supplementary Information 2.** Additional and diagnostic information for combined sample linear model explaining household conflicts with snow leopard conservation.

Potential predictor variables excluded from regression modelling due to equality of mean: livestock as primary source of financial income (t = 1.88, *p* = .060); tourism as primary source of financial income (t = 1.79, *p* = .074); other source as primary source of financial income (t = 0.43, *p* = .67). Potential predictor variables excluded from regression modelling due to a lack of correlation: household size (r = 0.33, *p* = .38); Sustainable Livelihoods index (r = 0.72, *p* = .077). This model had the highest significant R² change score (.020, p = <.001) out of the six models tested; variables excluded from final model due to lower R² change scores when included in successive models: number of household livestock killed by snow leopards (log¹º scale); cultivation as primary source of household income. **Diagnostics**

1. **Correlation matrix** check for multicollinearity – no predictors highly correlated with each other (>.9)

2. **Model summary** assumption of independent errors met as Durbin-Watson statistic between 1 and 3 (1.566)

3. **Coefficients** check for multicollinearity - VIF values are less than 10 and not substantially greater than 1

4. **Coefficients** check for multicollinearity – tolerance values are well above 0.2

5. **Casewise diagnostics** check for bias – 6.41% cases >2, 2.63% cases >2.5, 10 cases >3, indicating some bias in sample, hence bootstrapping

6. **Cook's distance** check for outliers – no values above 1.

7. **P-P plot** check for normality – line curving round diagonal indicates non-normality, hence bootstrapping.

**Supplementary Information 3.** Additional and diagnostic information for ACA sample linear model explaining household conflicts with snow leopard conservation.

Potential predictor variables excluded from regression modelling due to equality of mean: tourism as primary source of financial income (t = 1.15, *p* = .26); other source as primary source of financial income (t = 0.81, *p* = .42). Potential predictor variables excluded from regression modelling due to lack of correlation: household adult literacy rate (r = -0.038, *p* = .43); household size (r = 0.36, *p* = .45); Sustainable Livelihoods index (r = 0.91, *p* = .080). This model had the highest significant R² change score (.028, p = <.001) out of the four models tested; variables excluded from final model due to lower R² change scores when included in successive models: number of household livestock killed by snow leopards (log¹º scale); cultivation as primary source of household income.

**Diagnostics:**

1. **Correlation matrix** check for multicollinearity – no predictors highly correlated with each other (>.9)

2. **Model summary** assumption of independent errors met as Durbin-Watson statistic between 1 and 3 (1.566)

3. **Coefficients** check for multicollinearity - VIF values are less than 10 and not substantially greater than 1

4. **Coefficients** check for multicollinearity – tolerance values are well above 0.2

5. **Casewise diagnostics** check for bias – 7.87% cases >2, 3.37% cases >2.5, eight cases >3, indicating some bias in sample, hence bootstrapping.

6. **Cook's distance** check for outliers – no values above 1

7. **P-P plot** check for normality – line curving round diagonal indicates non-normality, hence bootstrapping.

**Supplementary Information 4**. Linear models explaining household livestock losses to snow leopards in previous 12 months for a combined sample, in ACA and in SNP.

| **Model** | **Variable** | ***b*** | ***SE B*** | ***Standardised b*** | ***p*** |
| --- | --- | --- | --- | --- | --- |
| **Combined**  R² = .430  N = 705 | **Constant** | .019  (-.007, .044) | .013 | --- | p = .18 |
|  | **Number of livestock owned by household (log¹º scale)** | -.022  (-.049, .001) | .013 | -.061 | p = .094 |
|  | **Total household members** | -.004  (-.010, .002) | .003 | -.040 | p = .17 |
|  | **Number of livestock lost by household (log¹º scale)** | .47  (.37, .55) | .044 | .69 | p = .001 |
| **ACA**  R² = .431  N = 445 | **Constant** | -.049  (-.11, .017) | .030 | --- | p = .10 |
|  | **Number of livestock owned by household (log¹º scale)** | -.035  (-.076, .003) | .020 | -.078 | p = .088 |
|  | **Number of livestock lost by household (log¹º scale)** | .49  (.39, .59) | .052 | .69 | p = .001 |
| **SNP**  R² = .388  N = 260 | **Constant** | .00  (-.029, .032) | .016 | --- | p = .99 |
|  | **Number of livestock owned by household (log¹º scale)** | .002  (-.024, .027) | .013 | .006 | p = .90 |
|  | **Total household members** | .00  (-.007, .006) | .003 | -.003 | p = .97 |
|  | **Number of livestock lost by household (log¹º scale)** | .38  (.26, .52) | .065 | .62 | p = .001 |

**Supplementary Information 5.** Additional and diagnostic information for combined sample linear model explaining household livestock losses to snow leopards.

Potential predictor variables excluded from regression modelling due to equality of mean: cultivation as primary source of household income (t = -0.38, *p* = .71); other types as primary source of household income (t = -0.86, *p* = .39). Potential predictor variables excluded from regression modelling due to a lack of correlation: household adult literacy rate (r = -0.058, *p* = .12); Sustainable Livelihoods Index score (t = 0.30, *p* = .46). This model had the highest significant R² change score (.32, p = <.001) out of the seven models tested; variables excluded from final model due to lower R² change scores when included in successive models: livestock as primary source of household income; tourism as primary source of household income; number of conflicts with snow leopard conservation; governance model.

**Diagnostics**

1. **Correlation matrix** check for multicollinearity – no predictors highly correlated with each other (>.9)

2. **Model summary** assumption of independent errors met as Durbin-Watson statistic between 1 and 3 (1.033)

3. **Coefficients** check for multicollinearity - VIF values are less than 10 and not substantially greater than 1

4. **Coefficients** check for multicollinearity – tolerance values are well above 0.2

5. **Casewise diagnostics** check for bias – 5.57% cases >2, 0.88% cases >2.5, two cases >3

6. **Cook's distance** check for outliers – no values above 1

7. **P-P plot** check for normality – line curving round diagonal indicates non-normality, hence bootstrapping.

**Supplementary Information 6**. Additional and diagnostic information for ACA sample linear model explaining household livestock losses to snow leopards.

Potential predictor variables excluded from regression modelling due to equality of means: tourism as primary source of household financial income (t = 1.22, *p* = .23); cultivation as primary source of household financial income (t = 1.44, *p* = .15); other types as primary source of household financial income (t = -1.25, *p* = .22). Potential predictor variables excluded from regression modelling due to a lack of correlation: household adult literacy rate (r = -0.069, *p* = .15); household size (r = 0.049, *p* = .30); Sustainable Livelihoods Index score (r = 0.004, *p* = .93). This model had the highest significant R² change score (.34, p = <.001) out of the four models tested; variables excluded from final model due to lower R² change scores when included in successive models: livestock as primary source of financial income; number of conflicts with snow leopard conservation.

**Diagnostics:**

1. **Correlation matrix** check for multicollinearity – no predictors highly correlated with each other (>.9)

2. **Model summary** assumption of independent errors met as Durbin-Watson statistic between 1 and 3 (1.041)

3. **Coefficients** check for multicollinearity - VIF values are less than 10 and not substantially greater than 1

4. **Coefficients** check for multicollinearity – tolerance values are well above 0.2

5. **Casewise diagnostics** check for bias – 5.39% cases >2, 0.89% cases >2.5, one case >3

6. **Cook's distance** check for outliers – no values above 1

7. **P-P plot** check for normality – line curving round diagonal indicates non-normality, hence bootstrapping.

**Supplementary Information 7**. Additional and diagnostic information for SNP sample linear model explaining household livestock losses to snow leopards.

Potential predictor variables not included in analysis due to small sample size in a category: livestock as primary source of household income (yes = 23); cultivation as primary source of household income (yes = 23); household conflicts with snow leopard conservation (yes = 10). Potential predictor variables excluded from regression modelling due to equality of mean: other types as primary source of household income (t = 1.45, *p* = .15); tourism as primary source of household income (t = -0.97, *p* = .33). Potential predictor variables excluded from regression modelling due to a lack of correlation: household adult literacy rate (r = 0.39, *p* = .53); Sustainable Livelihoods Index score (r = 0.50, *p* = .45). This model had the highest significant R² change score (.30, p = <.001) out of the three models tested.

**Diagnostics**

1. **Correlation matrix** check for multicollinearity – no predictors highly correlated with each other (>.9)

2. **Model summary** assumption of independent errors met as Durbin-Watson statistic between 1 and 3 (1.016)

3. **Coefficients** check for multicollinearity - VIF values are less than 10 and not substantially greater than 1

4. **Coefficients** check for multicollinearity – tolerance values are well above 0.2

5. **Casewise diagnostics** check for bias – 5.76% cases >2, 0.77% cases >2.5, one case >3

6. **Cook's distance** check for outliers – no values above 1

7. **P-P plot** check for normality – line curving round diagonal indicates non-normality, hence bootstrapping.

**Supplementary Information 8.** Sustainable livelihood index variables, sections and weighting.

| **Asset section** | **Section weighting** | **Variable name** | **Questionnaire data type** | **Index data type** | **Questionnaire number(s)** |
| --- | --- | --- | --- | --- | --- |
| Human | 20% | Adult literacy rate | Ratio | Ratio | 1.2.2 |
|  |  | School attendance rate | Ratio | Ratio | 1.2.3 |
|  |  | Medical treatment access | Ordinal | Categorical | 1.2.4 - 7 |
|  |  | Media access | Ordinal | Categorical | 1.2.8 - 11 |
| Natural | 20% | Grazing land access | Ordinal | Categorical | 1.3.1 |
|  |  | Livestock access | Continuous | Categorical | 1.3.2 - 6 |
|  |  | Cultivatable land access | Ordinal | Categorical | 1.3.7 |
|  |  | Natural products access | Ordinal | Categorical | 1.3.9 -13 |
|  |  | Water access | Ordinal | Categorical | 1.3.14 - 18 |
| Social | 20% | Formal organisation membership | Ordinal | Categorical | 1.4.1 - 9 |
|  |  | Political representatives access | Ordinal | Categorical | 1.4.10 - 12 |
| Physical | 20% | Fuel access | Ordinal | Categorical | 1.5.1 - 6 |
|  |  | Buildings access | Ordinal | Categorical | 1.5.7 - 10 |
|  |  | Transport access | Ordinal | Categorical | 1.5.11 - 17 |
| Financial | 20% | Household income | Categorical | Categorical | 1.6.1 |

**Supplementary Information 9.** Household questionnaire.

RA initials ___

**Human dimensions of snow leopard conservation:**

**Household questionnaire**

**Section 1 Household**

- 1. **Household location**

1.1.1 VDC

|  |
| --- |

1.1.2 Settlement

|  |
| --- |

1.1.3 Head of household’s name.

|  |
| --- |

.

1.1.4 House name/distinguishing features

|  |
| --- |

1.1.5 Adjacent landmark(s)

|  |
| --- |

- 1. **Sustainable Livelihoods Index: Human assets**

1.2.1 How many household members are there?

| Total | Adults (18+) | School age children (4-18) | Infants (<4) |
| --- | --- | --- | --- |
|  |  |  |  |

1.2.2 How many adult household members can read and write?

|  | N/A |  |
| --- | --- | --- |

1.2.3 How many household members of school age are in education?

|  | N/A |  |
| --- | --- | --- |

Which of the following types of medical treatment does your household have access to?

|  |  | Yes | No | N/A |
| --- | --- | --- | --- | --- |
| 1.2.4 | Self-administered traditional medicine |  |  |  |
| 1.2.5 | Self-administered ‘Western’ medicine |  |  |  |
| 1.2.6 | Visit to local clinic in PA |  |  |  |
| 1.2.7 | Visit to clinic outside of PA |  |  |  |

Which of the following types of media does your household have access to?

|  |  | Yes | No | N/A |
| --- | --- | --- | --- | --- |
| 1.2.8 | Newspaper |  |  |  |
| 1.2.9 | Radio |  |  |  |
| 1.2.10 | Television |  |  |  |
| 1.2.11 | Internet |  |  |  |

- 1. **Sustainable Livelihoods Index: Natural assets**
     1. Does your household have access to land for grazing animals?

| Yes |  | No |  | N/A |  |
| --- | --- | --- | --- | --- | --- |

How many of each type of livestock does your household own?

|  | Livestock | Number | N/A |
| --- | --- | --- | --- |
| 1.3.2 | Cattle |  |  |
| 1.3.3 | Sheep/goats |  |  |
| 1.3.4 | Horses/mules/donkeys |  |  |
| 1.3.5 | Yaks/yak hybrids |  |  |
| 1.3.6 | Other |  |  |

1.3.7 Does your household have access to land for agriculture/cultivation?

| Yes |  | No |  | N/A |  |
| --- | --- | --- | --- | --- | --- |

1.3.8 Are you able to sell any surplus agricultural products from your land?

| Yes |  | No |  | N/A |  |
| --- | --- | --- | --- | --- | --- |

Which of the following natural products does your household you have access to?

|  |  | Yes | No | N/A |
| --- | --- | --- | --- | --- |
| 1.3.9 | Fuelwood |  |  |  |
| 1.3.10 | Construction wood |  |  |  |
| 1.3.11 | Human food |  |  |  |
| 1.3.12 | Animal food |  |  |  |
| 1.3.13 | Medicinal plants |  |  |  |

Which of the following forms of water supply does your household have access to?

|  |  | Yes | No | N/A |
| --- | --- | --- | --- | --- |
| 1.3.14 | Spring |  |  |  |
| 1.3.15 | Well |  |  |  |
| 1.3.16 | Handpump |  |  |  |
| 1.3.17 | Outside tap |  |  |  |
| 1.3.18 | Inside tap |  |  |  |

- 1. **Sustainable Livelihoods Index: Social assets**

Is anyone in your household a member of any of the following formal groups/organisations?

|  |  | Yes | No | N/A |
| --- | --- | --- | --- | --- |
| 1.4.1 | Conservation committee |  |  |  |
| 1.4.2 | Village development committee |  |  |  |
| 1.4.3 | Tourism association |  |  |  |
| 1.4.4 | Microcredit group |  |  |  |
| 1.4.5 | Co-operative |  |  |  |
| 1.4.6 | Women’s group |  |  |  |
| 1.4.7 | School association |  |  |  |
| 1.4.8 | Youth group |  |  |  |
| 1.4.9 | Other |  |  |  |

Does your household have access to political representatives at the following levels?

|  |  | Yes | No | N/A |
| --- | --- | --- | --- | --- |
| 1.4.10 | Local |  |  |  |
| 1.4.11 | District |  |  |  |
| 1.4.12 | National |  |  |  |

- 1. **Sustainable Livelihoods Index: Physical assets**

Which of the following types of fuel sources does your household have access to?

|  |  | Yes | No | N/A |
| --- | --- | --- | --- | --- |
| 1.5.1 | Fuelwood |  |  |  |
| 1.5.2 | Cylinder gas |  |  |  |
| 1.5.3 | Kerosene oil |  |  |  |
| 1.5.4 | Electricity |  |  |  |
| 1.5.5 | Animal dung |  |  |  |
| 1.5.6 | Other |  |  |  |

Which of the following types of buildings does your household have access to?

|  |  | Yes | No | N/A |
| --- | --- | --- | --- | --- |
| 1.5.7 | Residential building |  |  |  |
| 1.5.8 | Joint tourist/residential building |  |  |  |
| 1.5.9 | Tourist building |  |  |  |
| 1.5.10 | Other building |  |  |  |

Which of the following forms of transport does your household have access to?

|  |  | Yes | No | N/A |
| --- | --- | --- | --- | --- |
| 1.5.11 | Foot |  |  |  |
| 1.5.12 | Animal |  |  |  |
| 1.5.13 | Bicycle |  |  |  |
| 1.5.14 | Bus/taxi |  |  |  |
| 1.5.15 | Aeroplane |  |  |  |
| 1.5.16 | Motorcycle |  |  |  |
| 1.5.17 | Other |  |  |  |

**1.6 Sustainable Livelihoods Index: Financial assets**

1.6.1 What was your total household income in the last 12 months (NR)?

| 0– 50,000 | 50,001– 100,000 | 100,001– 150,000 | 150,000- 200,000 | 200,001- 250,00 | >250,001 | Prefer not2say | N/A |
| --- | --- | --- | --- | --- | --- | --- | --- |
|  |  |  |  |  |  |  |  |

Which of the following types of financial income does your household have access to?

|  |  | Yes | No | N/A |
| --- | --- | --- | --- | --- |
| 1.6.2 | Livestock |  |  |  |
| 1.6.3 | Agriculture/cultivation |  |  |  |
| 1.6.4 | Wood |  |  |  |
| 1.6.5 | Other natural products |  |  |  |
| 1.6.6 | Tourism |  |  |  |
| 1.6.7 | Remittances |  |  |  |
| 1.6.8 | Savings |  |  |  |
| 1.6.9 | Loans |  |  |  |
| 1.6.10 | Other |  |  |  |

What are your most important sources of financial income as a household? RANK WITH 1 BEING THE MOST IMPORTANT.

|  |  | Rank |
| --- | --- | --- |
| 1.6.11 | Livestock |  |
| 1.6.12 | Agriculture/cultivation |  |
| 1.6.13 | Wood |  |
| 1.6.14 | Other natural products |  |
| 1.6.15 | Tourism |  |
| 1.6.16 | Remittances |  |
| 1.6.17 | Savings |  |
| 1.6.18 | Loans |  |
| 1.6.19 | Other |  |

**Section 2 Household conflict**

**2.1 Conflict with snow leopards**

2.1.1 What was the total number of livestock lost by the household in the last 12 months?

| Total number |  | N/A |  |
| --- | --- | --- | --- |

2.1.2 What were the numbers of each type of livestock lost by the household in the last 12 months?

| Cattle | Sheep/goats | Horses/mules | Yaks/yak hybrids | Other | N/A |
| --- | --- | --- | --- | --- | --- |
|  |  |  |  |  |  |

2.1.3 What were the most important reasons for these household livestock losses? RANK WITH 1 BEING THE MOST IMPORTANT.

| Disease | Weather | Snow leopards | Other predators | Theft | Accident | Other | N/A |
| --- | --- | --- | --- | --- | --- | --- | --- |
|  |  |  |  |  |  |  |  |

2.1.4 What was the total number of household livestock killed by snow leopards in the last 12 months?

| Total number |  | N/A |  |
| --- | --- | --- | --- |

2.1.5 What were the numbers of each type of household livestock killed by snow leopards in the last 12 months?

| Cattle | Sheep/goats | Horses/mules | Yaks/yak hybrids | Other | N/A |
| --- | --- | --- | --- | --- | --- |
|  |  |  |  |  |  |

2.1.6 Where were the main locations of these livestock killings by snow leopards? RANK WITH 1 BEING THE MOST IMPORTANT.

| High pastures | Low pastures | Barren land | Agriculture/  settlement | Scrubland | Other | N/A |
| --- | --- | --- | --- | --- | --- | --- |
|  |  |  |  |  |  |  |

2.1.7 Which were the main months when most of these livestock killings by snow leopards took place? RANK WITH 1 BEING THE MOST COMMON.

| Jan/  Feb | Feb/  Mar | Mar/  Apr | Apr/  May | May/  Jun | Jun  /July | Jul/  Aug | Aug/  Sep | Sep/  Oct | Oct/  Nov | Nov/  Dec | Dec/  Jan | Not sure | N/A |
| --- | --- | --- | --- | --- | --- | --- | --- | --- | --- | --- | --- | --- | --- |
| Magh | Falgun | Chaitra | Baisakh | Abhishek | Asar | Shrawan | Bhadau | Asoj | Kartik | Mangsir | Poush | --- | --- |
|  |  |  |  |  |  |  |  |  |  |  |  |  |  |

2.1.8 Did your household receive compensation for the livestock killed by snow leopards?

| Yes |  | No |  | Not yet |  | N/A |  |
| --- | --- | --- | --- | --- | --- | --- | --- |

2.1.9 Positive identification of snow leopard?

| Yes |  | No |  | N/A |  |
| --- | --- | --- | --- | --- | --- |

2.1.10 Positive differentiation between snow leopard and common leopard?

| Yes |  | No |  | N/A |  |
| --- | --- | --- | --- | --- | --- |

**2.2 Conflict with snow leopard conservation**

Has your household had a conflict with any of the following organisations involved in snow leopard conservation in the last 12 months and, if so, why was this?

|  |  | Yes | No | N/A |
| --- | --- | --- | --- | --- |
| 2.2.1 | Park management |  |  |  |
| 2.2.2 | Park management |  | |  |
| 2.2.3 | Local committee |  |  |  |
| 2.2.4 | Local committee |  | |  |

Has your household had a conflict with any of the following snow leopard conservation measures in the last 12 months and, if so, why was this?

|  |  | Yes | No | N/A |
| --- | --- | --- | --- | --- |
| 2.2.5 | Ban on the killing of snow leopards |  |  |  |
| 2.2.6 | Ban on the killing of snow leopards |  | |  |
| 2.2.7 | Ban on the killing of snow leopard prey |  |  |  |
| 2.2.8 | Ban on the killing of snow leopard prey |  | |  |
| 2.2.9 | Livestock compensation scheme |  |  |  |
| 2.2.10 | Livestock compensation scheme |  | |  |
| 2.2.11 | Corral construction |  |  |  |
| 2.2.12 | Corral construction |  | |  |
| 2.2.13 | Environmental education activities |  |  |  |
| 2.2.14 | Environmental education activities |  | |  |
| 2.2.15 | Limits on the collection of NTFPs |  |  |  |
| 2.2.16 | Limits on the collection of NTFPs |  | |  |
| 2.2.17 | Limits on the collection of wood |  |  |  |
| 2.2.18 | Limits on the collection of wood |  | |  |
| 2.2.19 | Other |  |  |  |
| 2.2.20 | Other |  | |  |

2.2.21 If other, please state what.

|  |
| --- |

**Supplementary Information 10.** Key informant interview sheet.

**Snow leopards and sustainability PhD:**

**Key Informant Interview Sheet**

**RA INITIALS:**

**INVESTIGATOR INITIALS:**

**Section 1 Socio-economic profile**

**1.1 Characteristics**

1.1.1 VDC:

1.1.2 Settlement:

1.1.3 Name/position/group:

**1.2 Human assets**

1.2.1 What are the standards of education like in the area?

1.2.2 Which types of medical treatment do household have access to?

1.2.3 Which types of media do households have access to?

**1.3 Natural assets**

1.3.1 What is livestock production like in the area?

1.3.2 What is agricultural production/cultivation like in the area?

1.3.3 Which (wild) natural products do households have access to?

1.3.4 Which forms of water supply do households have access to?

What is the current market value of the following livestock as sub-adults/adolescents (GIVE RANGE OF VALUES IF NECESSARY)?

| 1.3.5 | 1.3.6 | 1.3.7 | 1.3.8 |
| --- | --- | --- | --- |
| Cattle | Sheep/goats | Horses/mules/donkeys | Yaks/yak hybrids |
|  |  |  |  |

**1.4 Social assets**

1.4.1 Which formal groups/organisations do household have access to?

1.4.2 Which forms of political representation do households have access to?

**1.5 Physical assets**

1.5.1 Which types of fuel sources do households have access to?

1.5.2 Which types of buildings do households have access to?

1.5.3 Which forms of transport do households have access to?

**1.6 Financial assets**

1.6.1 What are household incomes (NR) like in the area?

1.6.2 Which sources of financial income do households have access to?

1.6.3 Which sources of financial income are most important?

**Section 2 Household conflict**

**2.1 Conflict with snow leopards**

2.1.1 What were the reasons for livestock losses in the area in the last 12 months?

2.1.2 What was the total number of livestock killed by snow leopards in the VDC in the last 12 months?

2.1.3 Where were the main locations of livestock killings by snow leopards?

2.1.4 Which were the main months when most of these livestock killings by snow leopards took place?

2.1.5 Did households receive compensation for the livestock killed by snow leopards?

**2.2 Conflict with snow leopard conservation**

2.2.1 Have households had conflict with organisations involved in snow leopard conservation in the last 12 months?

2.2.2 Have households had conflict with particular snow leopard conservation measures in the last 12 months?
